# Patterns of Nonemergent Visits to Different Healthcare Facilities on the Same Day: A Nationwide Analysis in Taiwan

**DOI:** 10.1155/2014/627580

**Published:** 2014-04-22

**Authors:** Meng-Hsuan Wu, Meng-Ju Wu, Li-Fang Chou, Tzeng-Ji Chen

**Affiliations:** ^1^Institute of Hospital and Health Care Administration, School of Medicine, National Yang-Ming University, Taipei 112, Taiwan; ^2^Faculty of Medicine, Semmelweis University, Budapest 1094, Hungary; ^3^Department of Public Finance, National Chengchi University, Taipei 116, Taiwan; ^4^Department of Family Medicine, Taipei Veterans General Hospital, Taipei 112, Taiwan

## Abstract

Doctor shopping is a common phenomenon in many countries. However, patterns of switching healthcare facilities on the same day were little known. The data were obtained from the longitudinal cohort datasets (LHID2010) of Taiwan's National Health Insurance Research Database in 2010. Of 1,000,000 persons of the cohort with 13,276,928 nonemergent visits, 185,347 patients had visited different healthcare facilities within one day, with a total of 672,478 visits and 337,260 switches between facilities in 329,073 patient-days. While 63.0% (*n* = 212,590) of all switches occurred between facilities of the same accreditation level, 14.1% (*n* = 47,664) moved from lower to higher level, and 22.8% (*n* = 77,006) moved in the opposite direction. In 33,689 switches, patients moved to the same specialty of another facility. In 48,324 switches, patients moved to another facility with the same diagnosis, and the most frequent diagnoses were diseases of the digestive system (11,148) and diseases of the respiratory system (10,393). In a densely populated country without strict referral regulation, a high percentage of Taiwanese people had the experience of visiting different healthcare facilities on the same day. The system of family physicians as personal doctors and gatekeepers to healthcare might ameliorate the harmful impact.

## 1. Introduction

Doctor shopping is a common phenomenon in many countries of the world [1–4] and has been also observed in Taiwan [5, 6]. Unlike healthcare systems in most developed countries, the National Health Insurance (NHI) in Taiwan does not have a formal gatekeeper system with restrictive regulations in referral, leading to annual number of ambulatory care visits per inhabitant as high as 13.4 and the incubation of doctor shopping behavior [5]. The analyses of doctor shopping in Taiwan have been applied to acute illness (upper respiratory tract infection) [7], chronic diseases (hypertension and type 2 diabetes mellitus) [8], and cancer [6]. In general, doctor shopping is operationally measured as a patient's visits to different physicians or healthcare facilities within a short period of time [5]. Two unique types of doctor shopping in Taiwan have been reported: (1) “one-stop visits” in which a patient pays visits to several specialties of the same healthcare facility within one day [5] and (2) “same-day visits” in which a patient pays visits to different healthcare facilities within one day. While the former phenomenon has been thoroughly analyzed with data mining technique [9], the latter deserves further detailed analysis.

The aim of the current study was to calculate the nationwide prevalence of one patient's visits to different healthcare facilities on the same day within Taiwan's NHI in 2010. More importantly, patterns of switching specialties and healthcare facilities on the same day would be analyzed. The findings might provide evidence for discussion in health policymaking.

## 2. Materials and Methods

The conduct of the study had been approved by the Institutional Review Board of Taipei Veterans General Hospital, Taipei, Taiwan (2013-01-005E).

### 2.1. Data Sources

The data were obtained from the longitudinal cohort datasets of 1,000,000 beneficiaries (LHID2010) of the National Health Insurance Research Database (NHIRD), managed by the National Health Research Institutes in Miaoli, Taiwan. These 1,000,000 persons were randomly sampled from 23,251,700 persons who had been insured under the NHI in 2010 (http://nhird.nhri.org.tw/date_cohort.htm). The claims belonging to the cohort were extracted from the whole database to form a specific dataset for research use. According to the NHIRD, the cohort did not differ from the population in the distributions of age, sex, and income subject to premium. In the current study, only the datasets of ambulatory visits in 2010 were used. One record of an ambulatory visit contains the patient's data (identification number, sex, birthday, visit date, and three diagnoses) and the provider's data (identification number of the healthcare facility and visited specialty). The original identification number of every beneficiary and healthcare facility has been encrypted in the NHIRD to protect privacy. The uniqueness of each identification number remains after encryption. The master file of healthcare facilities (HOSB) was used to know the level of accreditation.

### 2.2. Study Design

In 2010, the 1,000,000-person cohort had 15,431,528 ambulatory visit records. We calculated only those visits with physician consultations of western medicine (WM), dentistry, and traditional Chinese medicine (TCM). The visits to emergency departments were excluded from analysis.

The focus in the current study was on the patients who visited two or more healthcare facilities on the same day. The percentage of these patients in the cohort was computed and also stratified by age and sex. The number of visits involved in multiple visits to different healthcare facilities on the same day was additionally calculated.

Furthermore, we analyzed the patterns of multiple visits to different healthcare facilities on the same day by comparing the accreditation level of healthcare facility, type of visited specialty, and primary diagnosis in each visit with those in the ensuing visit. The sequence numbers of visits embedded in each patient's NHI chip card were used to clarify the direction of a patient's flow from one facility to another, from one specialty to another, and from one diagnosis to another. Four accreditation levels of healthcare facilities exist in Taiwan: academic medical center, metropolitan hospital, local community hospital, and physician clinic. The specialization in Taiwan includes 43 major specialties and 22 subspecialties. To simplify the analysis, the diagnosis in coding of International Classification of Diseases, Ninth Revision, Clinical Modification (ICD-9-CM), was grouped into the chapter of ICD-9-CM. Because a patient might move to and fro between healthcare facilities on the same day, we calculated only the first switch between any two facilities. Because a patient might visit more than one specialty in a facility, we compared only the last specialty of the outgoing facility with the first specialty of the incoming facility. The same method applied to the comparison between diagnoses.

### 2.3. Data Processing and Statistical Analysis

The Microsoft SQL Server 2012 was used for computing. The regular statistics were displayed. In calculating the percentages of patients in each age-sex group, the dominators were taken from the registry for 1,000,000 beneficiaries in 2010.

## 3. Results

**Figure 1:**
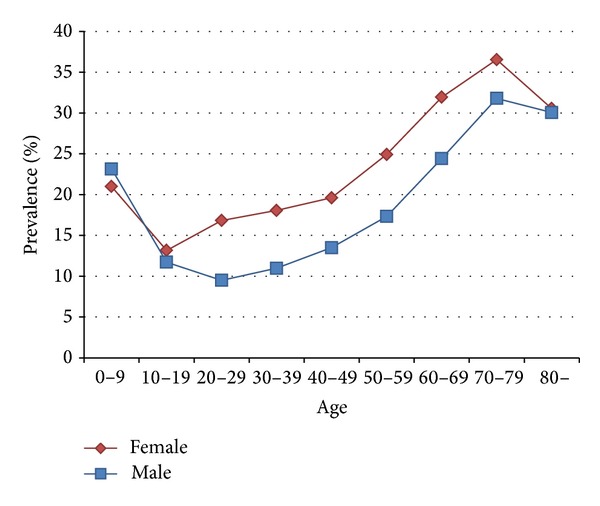
Age-sex distribution of patients with visits to different healthcare facilities within one day in the 1,000,000-person cohort in 2010.

Of 1,000,000 persons (507,577 women and 492,423 men) of the cohort with 13,276,928 valid visits, 185,347 patients (107,118 women and 78,229 men) had visited different healthcare facilities within one day in 2010, with a total of 672,478 visits in 329,073 patient-days. The average age was 42.6 ± 22.8 years (43.1 ± 21.7 in women and 41.9 ± 24.2 in men). Except in age group 0–9, women were more likely to visit different healthcare facilities within one day than men. Besides, children under 10 and old people of both sexes had a higher percentage of this phenomenon, with the peak in age group 70–79 (Figure 1). The overwhelming majority (97.6%, *n* = 321,258) of 329,073 patient-days with visits to different facilities within one day involved only two facilities, but in 30 cases a patient visited 6 facilities within one day (Table 1). On the other hand, 6.8% (*n* = 22,393) of 329,073 patient-days involved only one specialty; that is, a patient visited the same specialty of different facilities on the same day. In 2 extreme cases, a patient visited 7 specialties within one day (Table 1).

### 3.1. Distribution by Accreditation Level of Healthcare Facility

**Figure 2:**
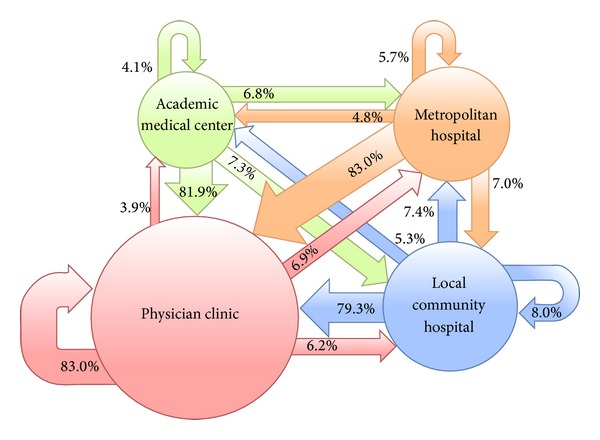
Flow of patients with visits to different accreditation level of healthcare facilities within one day in the 1,000,000-person cohort in 2010.

Of 329,073 patient-days with visits to different facilities within one day, there were 337,260 switches from one facility to the next facility. The largest group (*n* = 207,358) of these switches occurred between different physician clinics. While 63.0% (*n* = 212,590) of all switches occurred between facilities of the same accreditation level, 14.1% (*n* = 47,664) moved from a facility of a lower level to another of a higher level, and 22.8% (*n* = 77,006) moved in the opposite direction (Table 2) (Figure 2).

### 3.2. Distribution by Category of Specialty

Of 337,260 switches from one facility to the next facility within one day, the most frequent destinations by specialty were family medicine (45,524 times), TCM (42,102), and dentistry (39,231). In 33,689 switches, a patient moved to the same specialty of another facility and the most frequent destinations were family medicine (6,608), internal medicine (3,201), dentistry (2,160), pediatrics (2,141), and traditional Chinese medicine (1,839) (Table 3).

### 3.3. Distribution by Principal Diagnosis

Of 337,260 switches from one facility to the next facility within one day, the most frequent diagnoses at both outgoing and incoming facilities were diseases of the digestive system, diseases of the respiratory system, and diseases of the nervous system and sense organs. In 48,324 switches, a patient moved to another facility with the same diagnosis and the most frequent diagnoses were diseases of the digestive system (11,148) and diseases of the respiratory system (10,393) (Table 4).

## 4. Discussion

In our current study, we extended the traditional analyses to observe dynamic changes of patient visits in a large-scale, population-based dataset. Our study was purely descriptive. Although not qualitative in study design, our quantitative analysis did offer some clues.

Firstly, about one fifth of the Taiwanese had experience of switching healthcare facilities on the same day in a year and such visits accounted for 5.1% of all ambulatory care visits. Because there was neither formal referral system nor strict referral regulation within the NHI in Taiwan, the freedom of choosing healthcare facilities [10] and the low copayments [11, 12] might contribute to the occurrence of this phenomenon. The longer opening hours of healthcare facilities with walk-in registration [13] on the supply side might also play a role.

Secondly, of all switches on the same day, 16,308 (4.8%) occurred between hospitals. Usually the outpatient clinic of a hospital in Taiwan offered a broad spectrum of specialties so that the patients could consult several specialties in one visit (the so-called one-stop visit) [14]. For visits to different hospitals within one day, the patients might either search for a second opinion for the same illness or consult different hospitals for different illnesses. That is, a hospital might be good in some specialties and another in some other specialties. Because Taiwan is a densely populated island and most hospitals are located in cities, the patient could thus utilize the efficient transportation system to reach different hospitals in the shortest possible time.

Thirdly, with respect to specialty, 59,332 (17.6%) switches on the same day occurred between traditional Chinese medicine (TCM) and all specialties of western medicine (WM). Past studies had also revealed that patients in Taiwan might visit WM and TCM one after another within a short time [15, 16]. TCM can be traced back more than 2,000 years and still commonly used by people in China, Taiwan, Korea, and Japan [17]. More than 9,000 items of TCM herbal drugs are reimbursable within the NHI in Taiwan. Our finding highlights the importance that WM physicians should pay more attention to TCM drugs taken by patients to avoid drug interaction [5, 18, 19].

Furthermore, among switches on the same day with the same diagnosis, the most frequent diagnoses were diseases of the digestive system and diseases of the respiratory system. One of our earlier studies has revealed that these two kinds of diseases accounted for almost a half of ambulatory care visits in Taiwan and the number of visits with diseases of the respiratory system was twice that with diseases of digestive system [5]. However, in our current study, diseases of digestive system caused switches on the same day more frequently than diseases of the respiratory system. Perhaps diseases of digestive system bring more stress to patients. The reason deserves further analysis.

Our study with insurance claims of the NHIRD had some limitations. Firstly, the beneficiary's residence was unknown. The influence of location could not be studied. Secondly, we did not measure the distance between healthcare facilities that a patient visited on the same day. The traffic situation was not taken into consideration, either. Thirdly, the patient's complaints, symptoms, or reasons for consultations were not available. We could not know whether the switches between healthcare facilities were initiated by formal or informal referral from physicians, either.

## 5. Conclusion

In a densely populated country without strict referral regulation, a high percentage of Taiwanese people had the experience of visiting different healthcare facilities on the same day. It might represent the efficiency of Taiwan's NHI. However, in absence of communication between healthcare suppliers, the resulting duplicate examination and treatment might bring about problems of financial burden and patient safety. The system of family physicians as personal doctors and gatekeepers to healthcare might ameliorate the harmful impact [20].

## Figures and Tables

**Table 1:** Distribution of a patient's visits to different healthcare facilities within one day in the 1,000,000-person cohort in 2010, stratified by numbers of different healthcare facilities and specialties in a day.

	Number of patient-days	Percentage
Number of different healthcare facilities visited by a patient in a day		
6	6	0.0
5	17	0.0
4	320	0.1
3	7,472	2.3
2	321,258	97.6
Number of different specialties visited by a patient in a day		
7	2	0.0
6	8	0.0
5	53	0.0
4	555	0.2
3	10,609	3.2
2	295,453	89.8
1	22,393	6.8

**Table 2:** Distribution of a patient's switches (*n* = 337,260) between different healthcare facilities within one day in the 1,000,000-person cohort in 2010, stratified by accreditation level of healthcare facility.

	Incoming facilities					Total
	Academic medical center	Metropolitan hospital	Local community hospital	Physician clinic	Home care	
Outgoing facilities						
Academic medical center	**1,010**	1,672	1,788	20,178	0	24,648
Metropolitan hospital	1,660	**1,973**	2,419	28,719	0	34,771
Local community hospital	1,471	2,066	**2,249**	22,229	1	28,016
Physician clinic	9,736	17,362	15,369	**207,358**	0	249,825
Home care	0	0	0	0	**0**	0

Total	13,877	23,073	21,825	278,484	1	337,260

**Table 3:** Distribution of a patient's switches (*n* = 337,260) between different healthcare facilities within one day in the 1,000,000-person cohort in 2010, stratified by specialty.

	Specialty of incoming facility											Total	Percentage of switches between the same specialties
	Family medicine	Internal medicine	Pediatrics	Obstetrics and gynecology	Orthopedics	Otolaryngology	Ophthalmology	Dermatology	Dentistry	Traditional Chinese medicine	Other		
Specialty of outgoing facility													
Family medicine	**6,608**	4,053	1,488	1,710	1,846	2,692	5,906	2,471	5,290	5,696	7,067	44,827	14.7%
Internal medicine	4,470	**3,201**	880	1,460	1,308	2,125	4,560	2,137	3,666	4,202	5,746	33,755	9.5%
Pediatrics	1,155	748	**2,141**	558	322	1,309	2,456	1,226	2,950	1,542	1,578	15,985	13.4%
Obstetrics and gynecology	1,647	1,317	579	**1,750**	398	1,275	1,089	1,293	1,650	2,530	1,746	15,274	11.5%
Orthopedics	2,112	1,465	367	465	**722**	1,004	1,516	816	1,205	1,996	2,254	13,922	5.2%
Otolaryngology	2,580	1,951	1,128	1,263	824	**1,492**	4,592	2,642	4,403	2,995	3,519	27,389	5.4%
Ophthalmology	5,422	3,476	2,367	919	1,043	3,978	**1,230**	2,789	4,095	4,426	4,017	33,762	3.6%
Dermatology	2,187	1,671	1,042	1,079	526	2,196	2,733	**770**	2,664	3,084	2,489	20,441	3.8%
Dentistry	5,284	3,085	2,626	1,469	914	3,858	3,785	2,720	**2,160**	4,982	3,836	34,719	6.2%
Traditional Chinese medicine	4,468	2,963	1,424	1,691	1,162	2,320	3,406	2,505	4,299	**1,839**	4,112	30,189	6.1%
Other	9,591	7,021	2,073	2,357	2,681	5,287	6,499	4,053	6,849	8,810	**11,776**	66,997	

Total	45,524	30,951	16,115	14,721	11,746	27,536	37,772	23,422	39,231	42,102	48,140	337,260	6.5%

**Table 4:** Distribution of a patient's switches (*n* = 337,260) between different healthcare facilities within one day in the 1,000,000-person cohort in 2010, stratified by specialty principal diagnosis*.

		Diagnosis at incoming facility																		Total	Percentage of switches with the same diagnoses
	Ch01	Ch02	Ch03	Ch04	Ch05	Ch06	Ch07	Ch08	Ch09	Ch10	Ch11	Ch12	Ch13	Ch14	Ch15	Ch16	Ch17	V	Unknown		
Diagnosis at outgoing facility																					
Ch01	**787**	91	192	14	226	1,240	338	1,769	2,015	477	11	599	722	4	1	650	461	48	0	9,645	8.2%
Ch02	169	**801**	141	18	187	650	283	1,007	1,182	404	5	418	500	4	1	496	279	50	0	6,595	12.1%
Ch03	309	174	**725**	8	397	2,463	610	1,806	2,239	650	15	945	1,541	8	1	902	704	92	0	13,589	5.3%
Ch04	18	13	16	**23**	21	76	25	81	144	65	4	57	57	0	2	58	30	6	0	696	3.3%
Ch05	324	119	298	13	**1,444**	1,293	710	2,349	2,116	864	14	827	1,092	12	0	1,438	582	71	0	13,566	10.6%
Ch06	1,214	350	1,070	59	954	**4,041**	2,190	9,057	7,314	1,646	27	3,492	3,397	41	1	3,167	1,726	151	0	39,897	10.1%
Ch07	581	248	545	25	629	3,264	**1,592**	2,624	3,337	863	9	1,314	2,491	29	0	1,598	1,042	144	0	20,335	7.8%
Ch08	1,863	504	988	58	1,591	9,432	1,826	**10,393**	12,266	2,664	53	4,560	3,382	91	11	3,587	2,940	297	1	56,507	18.4%
Ch09	1,977	647	1,114	95	1,509	7,141	2,220	11,881	**11,148**	3,278	88	4,995	4,361	61	9	4,524	2,733	273	0	58,054	19.2%
Ch10	550	298	456	53	635	2,020	772	3,090	3,697	**2,763**	158	1,778	1,551	12	6	1,682	843	129	0	20,493	13.5%
Ch11	18	4	12	3	8	18	2	52	81	145	**233**	29	16	1	5	79	13	26	0	745	31.3%
Ch12	529	247	464	41	609	3,328	854	4,081	4,264	1,531	31	**2,961**	1,570	21	4	1,478	1,152	121	0	23,286	12.7%
Ch13	719	314	915	40	857	3,911	1,886	3,903	4,798	1,385	12	1,759	**5,249**	39	2	2,105	2,064	174	0	30,132	17.4%
Ch14	14	11	9	0	12	69	26	142	103	19	1	44	47	**77**	3	46	30	5	0	658	11.7%
Ch15	1	1	0	0	1	4	1	20	6	7	3	4	1	0	**4**	8	3	7	0	71	5.6%
Ch16	666	324	472	45	1,118	3,201	1,172	3,346	4,445	1,363	72	1,458	1,806	16	6	**2,803**	1,079	137	0	23,529	11.9%
Ch17	411	133	391	32	458	1,590	777	2,453	2,510	684	20	1,224	1,821	13	0	1,091	**3,255**	97	0	16,960	19.2%
V	83	41	68	3	79	216	133	347	436	188	34	192	248	7	19	211	171	**25**	0	2,501	1.0%
Unknown	0	0	0	0	0	0	0	1	0	0	0	0	0	0	0	0	0	0	**0**	1	

Total	10,233	4,320	7,876	530	10,735	43,957	15,417	58,402	62,101	18,996	790	26,656	29,852	436	75	25,923	19,107	1,853	1	337,260	14.3%

*Details of ICD-9-CM chapters in Table 5.

**Table 5:** 

Chapter	Diagnosis	Code range in ICD-9-CM
Ch01	Infectious and parasitic diseases	001–139
Ch02	Neoplasms	140–239
Ch03	Endocrine, nutritional, and metabolic diseases and immunity disorders	240–279
Ch04	Diseases of the blood and blood-forming organs	280–289
Ch05	Mental disorders	290–319
Ch06	Diseases of the nervous system and sense organs	320–389
Ch07	Diseases of the circulatory system	390–459
Ch08	Diseases of the respiratory system	460–519
Ch09	Diseases of the digestive system	520–579
Ch10	Diseases of the genitourinary system	580–629
Ch11	Complications of pregnancy, child birth, and the puerperium	630–677
Ch12	Diseases of the skin and subcutaneous tissue	680–709
Ch13	Diseases of the musculoskeletal system and connective tissue	710–739
Ch14	Congenital abnormalities	740–759
Ch15	Certain conditions originating in the perinatal period	760–779
Ch16	Symptoms, signs, and ill-defined conditions	780–799
Ch17	Injury and poisoning	800–999
V	Supplementary classification of factors influencing health status and contact with health services	V01–V82
